# Regulation of autophagy by non-coding RNAs in gastric cancer

**DOI:** 10.3389/fonc.2022.947332

**Published:** 2022-10-24

**Authors:** Zijian Wang, Jiarui Liu, Jingri Xie, Xingxing Yuan, Bingyu Wang, Wenjuan Shen, Yang Zhang

**Affiliations:** ^1^ Graduate College, Heilongjiang University of Chinese Medicine, Harbin, China; ^2^ College of Life Science and Technology, Guangxi University, Nanning, China; ^3^ Department of Gastroenterology, The First Affiliated Hospital of Heilongjiang University of Chinese Medicine, Harbin, China; ^4^ Department of Gastroenterology, Heilongjiang Academy of Traditional Chinese Medicine, Harbin, China; ^5^ Department of Gynaecology, The First Affiliated Hospital of Heilongjiang University of Chinese Medicine, Harbin, China

**Keywords:** autophagy, gastric cancer, non-coding RNA, tumorigenesis, chemoresistance

## Abstract

Autophagy is a conserved cellular self-digesting process that degrades obsoleting proteins and cellular components and plays a crucial role in the tumorigenesis, metastasis, and drug resistance of various tumors such as gastric cancer (GC). As a hotspot in molecular biology, non-coding RNAs (ncRNAs) are involved in the regulation of multiple biological processes, such as autophagy. Increasing evidence indicate that various ncRNAs exert double roles in the initiation and progression of GC, either serve as oncogenes or tumor suppressors. Recent studies have shown that some ncRNAs could modulate autophagy activity in GC cells, which would affect the malignant transformation and drug resistance. Whether the function of ncRNAs in GC is dependent on autophagy is undefined. Therefore, identifying the underlying moleculr targets of ncRNAs in autophagy pathways and the role of ncRNA-regulated autophagy in GC could develop new treatment interventions for this disease. This review summarizes the autophagy process and its role in GC, and the regulatory mechanisms of ncRNAs, as well as focuses on the dual role of ncRNAs-mediated autophagy in GC, for the development of potential therapeutic strategies in GC patients.

## Introduction

Gastric cancer (GC) is a gastrointestinal tumor accounting for nearly 15% of cancer-related deaths in the world, with high morbidity and high-grade malignancy (1). Surgical resection is a curative treatment for patients with early GC, whereas systemic chemotherapy provides an optimal therapeutic strategy for patients who have developed local or distant metastasis (2). Unfortunately, GC tumors increasingly become resistant to chemotherapy because of the intrinsic or acquired mechanisms of chemoresistance, which contributes to tumor metastasis and local recurrence (3). Autophagy is an evolutionarily conserved intracellular self-digesting process by which misfolded proteins and damaged organelles are degraded to regulate cell homeostasis (4). This process has become a hot topic in cancer research within recent years due to its involvement in tumorigenesis, metastasis, and chemoresistance (5). During tumor progression or chemotherapy-induced stress, obsolete organelles and useless proteins are recycled by autophagy to foster cancer cell growth and chemoresistance; conversely, removal of oncogenic substances *via* autophagy blocks tumorigenesis (6). Thus, the role of autophagy as a “friend” or “foe” in GC progression and chemoresistance is still controversial (7). It should be noted that autophagy comprises multiple autophagy-related genes (ATGs) and complexes that are indispensable to the formation of autophagosome, and that autophagy is intricately regulated by a variety of signaling pathways (8). Autophagic components and autophagy-related signaling molecules can be further modulated by non-coding RNAs (ncRNAs) at various levels, from transcriptional regulation to post-translational protein modification (9). Recent findings indicate that ncRNA-mediated autophagy is associated with malignant behaviors like proliferation, metastasis and chemoresistance that is extremely important in cancer development (10). Whether the dual role of autophagy in GC is determined by ncRNAs is still vague. Thus, it is crucial to clarify the regulative mechanisms of ncRNA on autophagy of GC. This review concisely summarizes the formation, signal regulation of autophagy and its paradoxical effects in GC, as well as the regulatory mechanisms of ncRNAs in the gene expression and protein modification. It emphasizes the latest research progression on the role of ncRNAs-regulated autophagy in GC, and discusses the effects of drug-mediated autophagy through regulating ncRNAs on GC treatment.

## The process, pathways and roles of autophagy

**Figure 1:**
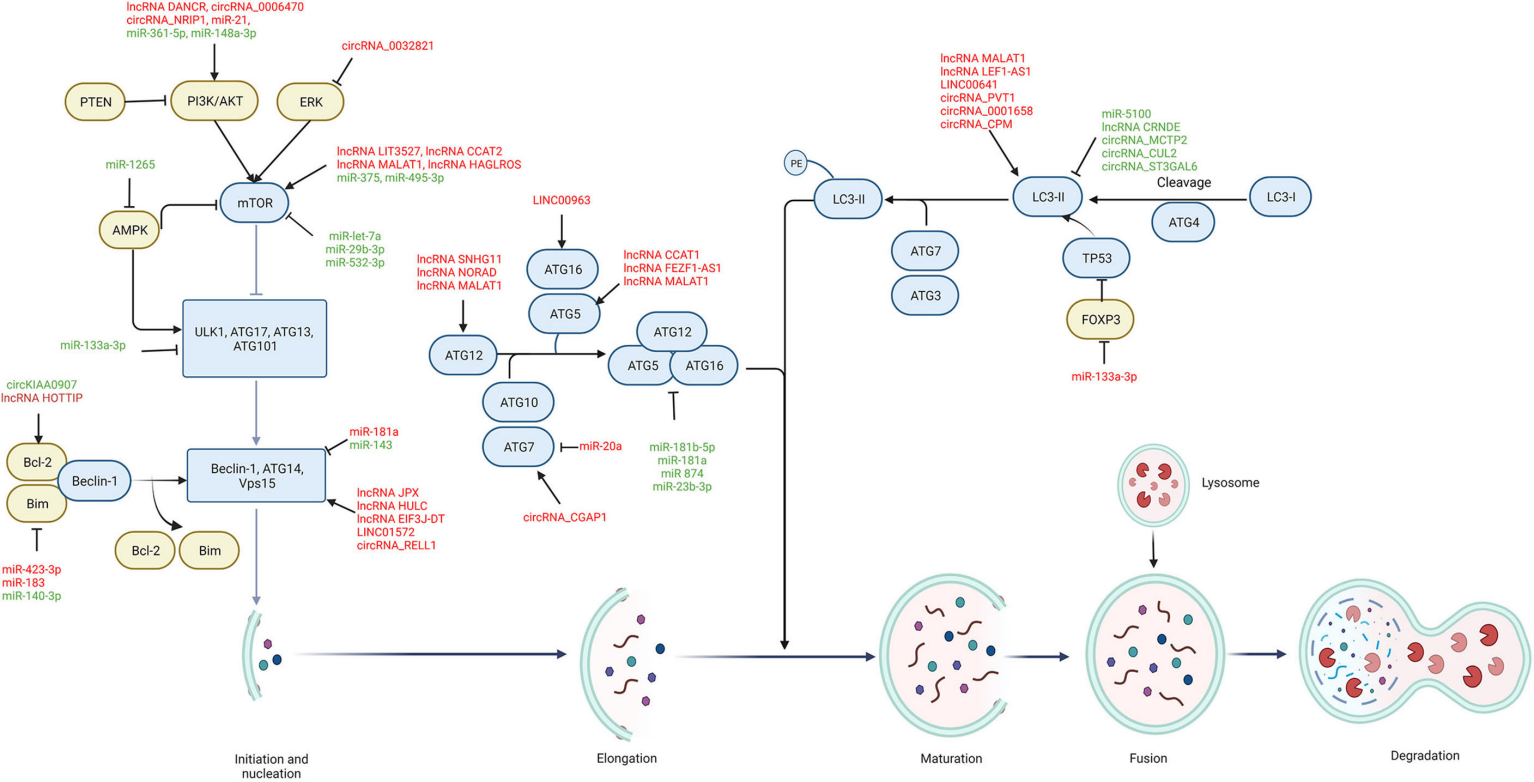
The regulatory role of ncRNAs on autophagy process in GC. Autophagic components including autophagy-related signaling pathways (yellow) and autophagic proteins (blue) are regulated by ncRNAs during each stage of autophagy, including initiation and nucleation, elongation, maturation, fusion, and degradation. Oncogenic ncRNAs (red) are highly expressed in GC and promote cancer progression and chemoresistance by modulating autophagic components, while tumor-suppressive ncRNAs (green) are downregulated in GC and their upregulation can suppress GC progression and chemoresistance through autophagy modulation. → indicates a promoting effect and ⊥ indicates an inhibitory effect.

Autophagy can be generally classified into macroautophagy, microautophagy, and chaperone-mediated autophagy (11). Owing to its extensive and crucial roles in current research, macroautophagy is referred to as autophagy in this review. Cellular conditions like nutrient deficiency, hypoxia, and inflammation, can trigger autophagy signals in the affected cells (12). The process of autophagy consists of five steps: initiation and nucleation, elongation, maturation, fusion, and degradation. Each step of autophagy is regulated by ATGs and molecular complexes (**Figure 1**) (13). The inactivation of mammalian target of rapamycin (mTORC) complex induces the activation of a protein complex, composing of unc-51 like autophagy activating kinase (ULK1), ATG17, ATG13, and ATG101, which is recruited to form a pre-autophagosomal structure (PAS) that initiates the autophagy process. Inhibition of the mTOR/ULK1 signaling pathway activates autophagy signal in GC cells (14). This activated ULK1 complex phosphorylates the autophagy and beclin-1 regulator (AMBRA) protein that activates the class III phosphatidylinositol 3-kinase (PI3K) complex consisting of PI3KC3, beclin-1, ATG14 and vacuolar protein sorting 15 (VPS15), and produces phosphatidylinositol 3-phosphate (PI3P) at the endoplasmic reticulum (ER) (15, 16). PI3P subsequently attracts zinc-finger FYVE domain-containing protein 1 (ZFYVE1, also called DFCP1) and WD repeat domain phosphoinositide-interacting protein (WIPI2), which bind the ATG16L1 protein and make it accessible to recruit the ATG16L1/ATG5/ATG12 protein complex to the PAS (17). Reducing the expression of ATG16L1 in GC cells blocks the formation of ATG16L1/ATG5/ATG12 complex and thus suppress the occurrence of autophagy (18). This protein complex also combines with the ATG3/ATG7 complex to promote the binding of LC3-II, derived from the cleavage of microtubule associated protein light chain 3 (LC3) by cysteine protease ATG4, with phosphatidylethanolamine (PE) to form PE-conjugated LC3-II, which elicits the membrane sealing to form a mature autophagosome, and further to bind with cargo receptors such as p62 to select proteins or organelles (19). Then, the autophagosome fuses with the lysosome to form autolysosome. As the fusion completes, the autophagosome substances will be degraded and released from the autolysosome. These products of decomposition can be recycled for cellular anabolism and growth.

Several signaling pathways are demonstrated to be involved in the regulation of autophagy. The inactivation of the mTOR signaling pathway is the central step to induce autophagy, which can be regulated by various signaling pathways, such as PI3K/AKT, adenosine monophosphate-activated protein kinase (AMPK), mitogen activated kinase-like protein (MAPK), and phosphatase and tensin homolog (PTEN) pathways (20). The PI3K phosphorylates phosphatidylinositol-4,5-bisphosphate to produce PI3P that induces Akt phosphorylation, which activates mTOR and results in the inhibition of autophagy in GC cells (21). Besides, cellular energy deficiency increases the level of AMPK that phosphorylates ULK1 to directly activate autophagy, as well as inactivates mTOR to indirectly induce autophagy (22). As an important regulating protein of the autophagic pathway, Bcl-2 binds to beclin-1 and thus blocks the formation of autophagosome. The c-Jun N-terminal kinase (JNK), a subtype of MAPK, phosphorylates Bcl-2, contributing to the dissociation of beclin-1 and the subsequent induction of autophagy (23). However, extracellular signal-regulated kinase (ERK), another subtype of MAPK, can be phosphorylated to block the Tuberous sclerosis complex 1 and 2 (TSC1/TSC2) complex and activate mTOR, thus leading to the suppression of autophagy. In addition, the presence of PTEN inhibits the PI3K/AKT signaling pathway (24). Consequently, inactivation of the AKT signaling pathway by PTEN facilitates autophagy activity.

Autophagy is believed to play dual roles in tumor progression that acts as a suppressor at early stages but as a promoter at the advanced stages of GC (6). For the tumor-inhibiting role, autophagy mitigates tumor initiation by removal of oncogenic substances and strengthens tumor immunosurveillance by interacting with factors involved in innate or adaptive immunity; moreover, excessive autophagy causes cellular energy depletion and ultimately leads to cell death, which is known as autophagic cell death (25). On the other hand, autophagy functions as a “recycling” process that digests useless and toxic components, and produces nutrients and essentials to maintain cell homeostasis, by which GC cells also survive under stress conditions such as hypoxia and chemotherapy (26).

## Regulatory mechanisms of NcRNAs

NcRNAs mainly contain microRNA (miRNA), long non-coding RNA (lncRNA) and circular RNA (circRNA). MiRNAs are a kind of small endogenous, single-stranded ncRNAs with a length of 19–25 nucleotides. By pairing with the complementary sequences at the 3′-UTR of mRNAs of target genes, miRNAs suppress mRNAs translation or initiate their degradation (27). Depending on the degree of complementarity between miRNA and the target mRNA, different outputs can occur. The mRNA undergoes endonucleolytic cleavage when the complementarity is perfect, but, if the complementarity is not total, the target mRNA is translationally repressed, through interference with polyribosomes or by sequestering in cytoplasmic P-bodies (28). Thus, miRNAs function as master regulators that control the expression of genes, and are involved in the occurrence and progression of human diseases, such as tumors, neurodegenerative diseases, autoimmune diseases, and endocrine disorders (29). Of interest, the role of miRNAs in tumorigenesis is double-edged: acting as tumor suppressors by blocking the translation of mRNA of target genes that mediate differentiation, invasion, and malignance; conversely, playing oncogenic role *via* triggering the degradation of mRNA of tumor suppressor genes (30). Numerous studies have investigated the potential roles of miRNAs as a diagnostic or prognostic marker for cancer, and, as promising therapeutic agents to tackle cancer (31, 32).

LncRNAs are a class of transcripts of over 200 nucleotides, accounting for 80–90% of all ncRNAs, and no or limited protein-coding potential (33). The regulatory mechanisms of lncRNAs are determined by their structure. On the primary structure level, the functional activity of lncRNAs relies on Watson-Crick base pairing, promoting direct interactions with other RNA molecules. The secondary structures are shaped by base-pairing or ribose backbone interactions, that allow the formation of higher-order configurations with helices and hairpin loops. A third alternative mechanism of stabilization is circularization, leading to the formation of the circRNAs (34). There are three well-characterized action mechanisms of lncRNAs (1): interacting with DNA, chromatin-modifying enzymes, and transcription factors to regulate genetic transcription (2); sponging miRNAs from their target mRNAs or direct binding to mRNAs to influence translation, also known as a competing endogenous RNAs (ceRNAs) mechanism (3); serving as scaffolds for proteins to block their functions (35). LncRNAs have been demonstrated to widely participate in various physiological and pathological processes (36, 37). Some overexpressed lncRNAs in cancers are reported to promote tumor growth by regulating angiogenesis, migration and metastasis, while other downregulated lncRNAs are regarded as safeguards against cancer progression by inhibiting cell proliferation, inducing apoptosis, maintaining genomic stability, or promoting tumor suppressor expression (38).

CircRNAs are a kind of single-stranded covalently RNA molecules that form a closed loop through the link between 5’ and 3’ terminal nucleotide sequences, with the length of 100nt ~ over 4 kb (39). Thus, comparing with linear transcripts such as miRNAs and lncRNAs, circRNAs are more stable owing to lacking of 3ʹ and 5ʹ ends, suggesting a huge potential for diagnostic or prognostic markers of various diseases (40, 41). The exact action models of circRNAs are still unsolved, but they are believed to affect different biological processes in two manners: acting as sponges directly binding to miRNAs to prevent them from exerting biological functions; moreover, functioning as scaffolds for the protein complex assembly to suppress its functions (42). Moreover, circRNAs act as transcription modulators. It is assumed that circRNAs are nuclear limited, which is similar to the observation of nuclear limitation of linear RNAs containing conserved introns and forms a large number of posttranscriptional modulators. Thus, further suppression of circRNAs leads to a decrease in the expression of their parental genes (43). CircRNAs have become a research hotspot because of their association with disease progression, particularly in cancer (44, 45). As circRNAs regulate both the expression of oncogenes and tumor suppressors, they also play dual role in tumorigenesis (46).

**Figure 2:**
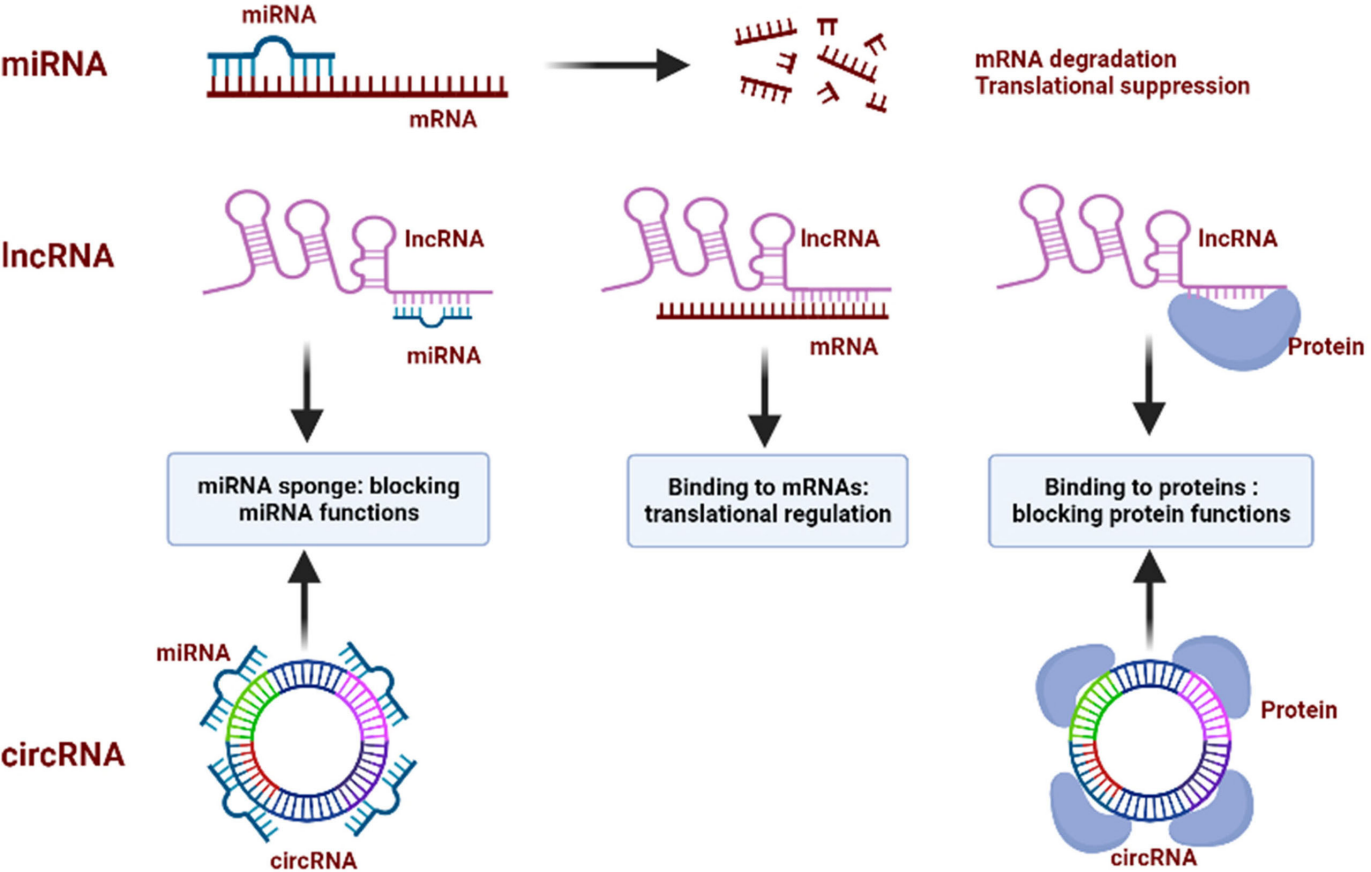
Regulatory mechanisms of ncRNAs. miRNAs bind to the target mRNAs and inhibit the function of mRNAs by inducing its degradation and blocking the translational process; lncRNAs sponge miRNAs to block their functions, bind to mRNAs to regulate their translation, and interact with proteins to repress their functions; circRNAs sponge miRNAs to block their functions, and bind to proteins to block their functions.

In conclusion, miRNAs directly target mRNAs to influence the gene translational process. Both lncRNAs and circRNAs can act as ceRNAs to sponge miRNAs, thus regulating the miRNAs-mediated gene epigenetic regulation. They also directly bind to proteins, suppressing proteins’ functions and blocking downstream signaling pathways (**Figure 2**). Dysfunction of ncRNAs is frequently emerged in cancer where ncRNAs function as both oncogenes and tumor suppressors. In this context, ncRNAs are capable of affecting various cellular processes, including autophagy, as they can regulate the expression of ATGs and autophagy-related signaling proteins. Thus, it can be speculated that the role of autophagy in tumors is can be further regulated by ncRNAs.

## Roles of autophagy-regulating NcRNAs in GC

A total of 54 ncRNAs reported from experimental and clinical studies of GC are included to discuss their roles in regulating autophagy process in GC progression and chemoresistance (**Tables 1**–**4**). Mechanically, these autophagy-regulating ncRNAs modulate cellular autophagy by regulating ATGs and autophagy-related signaling pathways (**Figure 1**).

**Table 1:** NcRNAs serve as tumor promoters in GC.

NcRNA	Expression	Targets	Autophagy	Outcome	Experimental model	Ref.
miR-133a-3p	Upregulated	FOXP3↓→TP53↑, LC3-II↑	Activated	Increase the proliferation of GC cells	AGS and MGC803 cells	(47)
miR-423-3p	Upregulated	Bim↓→beclin-1↑	Activated	Promote cell proliferation, migration, and invasion	BGC823, MGC803, SGC7901, and MKN45 cells; human serum and GC tissues	(48)
miR-181a	Upregulated	MTMR3↓→PI3P↓, LC3-II↓	Inhibited	Promote proliferation, colony formation, migration and invasion, and inhibit apoptosis	AGS cells	(49)
miR-183	Upregulated	UVRAG↓→Bax/Bcl-2↓, LC3-II↓	Inhibited	Enhance cell survival and desensitize cells to starvation-induced apoptosis	MKN28 cells	(50)
miR-20a	Upregulated	Beclin-1↓, ATG5↓, ATG7↓	Inhibited	Reduce cell apoptosis	SGC7901, MKN45 cells; human GC tissues	(51)
LncRNA LIT3527	Upregulated	mTOR↑→LC3-II↓	Inhibited	Promote proliferation, and lung metastasis	AGS, MKN45, MKN74, HGC27, MGC803 cells; human GC tissues	(52)
LncRNA CCAT2	Upregulated	mTOR↑→beclin-1↓	Inhibited	Promote proliferation and inhibit apoptosis	BGC823 cells	(53)
LncRNA MALAT1	Upregulated	mTOR↑→p62↑	Inhibited	Facilitate IL-6-activated normal to cancer-associated fibroblast	MKN45, MGC-803 cells; human GC tissues	(54)
LncRNA MALAT1	Upregulated	miR-204↓→ LC3-II ↑, TRPM3↑	Activated	Promote proliferation	MKN45, CTC141 and CTC105 cells; human GC tissues	(55)
LncRNA JPX	Upregulated	miR-197↓→ beclin-1↑, p62↓	Activated	Increase cell activity, migration and invasion	NCIN87 and MKN45 cells; human GC tissues	(56)
LncRNA HAGLROS	Upregulated	miR-100-5p↓→mTOR↑ → ATG9↓	Inhibited	Promote proliferation, invasion, migration	SGC7901, BGC823, HGC27, MGC803 and AGS cells; human GC tissues	(57)
LncRNA DANCR	Upregulated	miR-194↓- AKT2↑→beclin-1↓	Inhibited	Increase cell viability, reduce apoptosis	SGC7901, MGC803, and NCIN87 cells; human GC tissues	(58)
LncRNA CCAT1	Upregulated	miR-140-3p↓→ ATG5↑	Activated	Promote proliferation, migration, invasion	AGS, MKN45 cells; nude mice	(59)
LncRNA SNHG11	Upregulated	miR-483-3p/miR-1276↓→ATG12↑	Activated	Facilitate proliferation, stemness, migration, invasion, and ETM.	SGC7901, BGC823, MGC803, MKN45, HGC27, and AGS cells; human GC tissues	(60)
LncRNA LEF1-AS1	Upregulated	miR-5100/DEK/AMPK/mTOR↑ →LC3-II↑, p62↓	Activated	Accelerates metastasis and growth	HGC-27, MGC80-3, SGC7901 and AGS cells; nude mice	(61)
CircRNA_0032821	Upregulated	MEK1/ERK1/2 ↓→p62↑	Inhibited	Promote proliferation, ETM, migration and invasion	AGS, HGC27, MKN74, MKN1 cells	(62)
CircRNA_0006470	Upregulated	miR-27b-3p↓→PI3KCA↑→beclin-1↓, p62↑	Inhibited	Promote proliferation, migration, and invasion	AGS cells	(63)
CircRNA_UBE2Q2	Upregulated	miR-370-3p↓→STAT3↑→p62↑, Bcl-2↑	Inhibited	Promote proliferation, migration, invasion, glycolysis, metastasis	BGC823, SGC7901, MKN45, MGC803, HGC27 cells; human GC tissues	(64)
CircRNA_NRIP1	Upregulated	miR-149-5p↓→AKT1↑→ULK↑, p62↑	Inhibited	Promote proliferation, migration, invasion	BGC823, SGC7901, MGC803, MKN45, HGC27, and AGS cells; human GC tissues	(65)
CircRNA_0001658	Upregulated	miR-182↓→Rab-10↑→LC3-II↑, p62↓	Activated	Promote cell viability and reduce apoptosis	AGS and HGC27 cells	(66)

↑, upregulation; ↓, downregulation.

### Tumor promoter

MiRNA molecules have been proved to directly regulate target genes to promote cancer cell proliferation in which autophagy is involved. For example, the miR-133a-3p/FOXP3 axis, playing an oncogenic role in GC cells, triggers the proliferation and autophagy *via* suppressing the expression of TP3, a tumor suppressor gene (47). Kong et al. illuminated that miR-423-3p, which indicated a poor prognosis in GC, was highly expressed in GC tissues and promoted cancer progression; however, this effect was blocked by knockdown of the ATG7, suggesting the oncogenic role of miR-423-3p *via* beclin-1-mediated autophagy (48). These findings indicate that oncogenic miRNAs trigger GC progression *via* inducing autophagy. However, miR-181a, highly expressed in both human GC tissues and cell lines, represses autophagy and thus leads to elevated migration and invasion *via* decreasing the expression of myotubularin related protein 3 (49), which contains PI3P phosphatases proteins that is required for the initiation of autophagy (67). Similarly, the ultraviolet radiation resistance-associated gene (UVRAG) is demonstrated to interact with Bcl-2 to further induce autophagy and GC cell death; moreover, miR-183 attenuates starvation-induced autophagy and apoptosis in GC cells by targeting the 3′-UTR region of UVRAG, suggesting an autophagy-related oncogenic role of miR-183 (50). As an oncogene, miR-20a is upregulated in GC and reduces both autophagy and apoptosis induced by the METase/SNHG5 axis (51). These results imply that oncogenic miRNAs also induce the malignant phenotype of GC *via* inhibiting both apoptosis and autophagy. H. pylori infection is believed to be the main risk factor for GC. Of interest, these bacteria induce the expression of miRNAs in host cells to facilitate GC progression *via* modulating autophagy. For instance, CagA released from H. pylori increases the expression of miR-543, which aggravates cell proliferation, migration, and invasion by impairing SIRT1-induced autophagy (68). Likewise, H. pylori-induced miR-30d overexpression in GC restrains autophagy to mediate H. pylori persistence and cancer progression through inhibiting the expression of ATG5, ATG12, and beclin-1 (69). Therefore, the infection-induced upregulation of miRNAs blocks autophagy and thus prevents H. pylori from autophagy-mediated clearance.

LncRNAs also promote GC progression by regulating autophagy. It was reported that lncRNA LIT3527 was highly expressed in GC, and its depletion drove cell autophagy and apoptosis *via* inhibiting AKT/ERK/mTOR signaling pathway, along with reduced proliferation, migration and lung metastasis of GC cells (52). Analogously, overexpressed lncRNA CCAT2 is observed in GC tissues; moreover, silencing of CCAT2 mediates apoptosis and autophagy, as well as the proliferation of BGC823 cells by suppressing the PI3K/mTOR activity (53). Intriguingly, upregulated lncRNA MALAT1 in GC cells protects IL-6 from autophagic degradation and thus aggravates IL-6-activated cancer-associated fibroblast conversion. Further mechanistic investigations explain that MALAT1 competitively interacts with ELAVL1 to destabilize PTEN mRNA because ELAVL1 bind a greater fraction of MALAT1 mRNA than PTEN mRNA, therefore blocking autophagy by activating the AKT/mTOR signaling pathway (54). Also, MALAT1 is demonstrated to function as a decoy of miR-204 to enhance GC cell proliferation but attenuate autophagy through downregulating the expression of LC3-II (55). Likewise, lncRNA JPX targets miR-197 to suppress beclin-1 expression while promoting p62 expression in GC cells, thus accelerating GC progression by regulating autophagy (56). New evidence shows that lncRNA HAGLROS, an indicator of a poor prognosis in GC patients, strengthens GC cell proliferation and maintains the malignant phenotype by inhibiting autophagy through competitively interacting with miR-100-5p to further activate mTORC1 and alleviate the expression of ATG9 (57). Besides, KLF5-induced lncRNA DANCR overexpression in GC elevates cancer cell viability while impeding autophagy through the miR-194/AKT2 axis (58). These results indicate that oncogenic lncRNAs mediate GC progression by suppressing autophagy through direct regulating autophagy-related pathways and acting as a sponge of miRNAs to indirectly inhibit the expression of ATGs. However, lncRNA CCAT1 promotes GC cell proliferation, migration and invasion, accompanied by increased autophagy activity by sponging miR-140-3p to restore the expression of ATG5 (59). High expression of lncRNA SNHG11 in GC also aggravates oncogenic autophagy by upregulating ATG12 expression through the miR-483-3p/miR-1276 axis (60). Similarly, overexpressed lncRNA LEF1-AS1 promotes the autophagy and malignant phenotype of GC by the miR-5100/DEK/AMPK/mTOR axis (61). Thus, lncRNAs also promote GC progression by inducing autophagy through sequestering miRNAs and enhancing ATGs expression.

Also, circRNAs play an oncogenic role in GC. It was reported that high circRNA_0032821 expression in patients with GC was associated with lymph node metastasis and poor prognosis. Further investigations unveiled that circRNA_0032821 facilitated GC cell proliferation, migration and invasion, but alleviated autophagy through activating the MEK1/ERK1/2 signaling pathway (62). Similarly, the elevated expression of circRNA_0006470 GC cells improved cell proliferation and migration through targeting miR-27b-3p to suppress autophagy. Mechanistic evaluation showed that circRNA_0006470 reduced the expression of LC3-II and beclin-1 but increased the expression of p62 by sponging miR-27b-3p to activate ROR1 and PI3KCA (63). Besides, the oncogenic circRNA_UBE2Q2 promoted glycolysis for GC cell invasion and metastasis through activating the miR-370-3p/STAT3 pathway (64). The AKT1/mTOR signaling pathway is also activated by circRNA_NRIP1, which inhibits autophagy but promotes GC progression by sponging miR-149-5p, indicating an underlying therapeutic target for GC (65). These studies suggest that oncogenic circRNAs in GC suppress autophagy by regulating autophagy-related pathways *via* targeting miRNAs. However, a recent study has reported that circRNA_0001658 promotes autophagy and GC cell viability through miR-182/Rab-10 axis (66).

It can be concluded that oncogenic ncRNAs are upregulated in GC tissues and promote malignant progression *via* activating or inhibiting autophagy. It is generally acknowledged that enhanced autophagy evokes GC progression in most cases owing to its cytoprotective feature (6). However, most oncogenic ncRNAs are related to low levels of autophagy, especially when ncRNAs function through regulating autophagy-related signaling pathways, including mTOR, PI3K, AKT, and ERK (**Table 1**). One explanation may be that oncogenic ncRNAs target these signaling pathways to block autophagic cell death. Hence, silencing these ncRNAs or targeting these downstream molecules may suppress tumorigenesis and progression by inducing autophagic cell death. Besides, it should be note that autophagy has been verified to interact with multiple biological processes associated with cancer progression, such as apoptosis, oxidative stress, DNA damage and repair (70, 71). Indeed, most ncRNAs exert their oncogenic role by inhibiting apoptosis and autophagy (**Table 1**). Thus, oncogenic ncRNAs in GC may mainly regulate malignant transformation processes with an additional autophagy suppression. It can be assumed that oncogenic ncRNAs-regulating autophagy in GC progression is complex due to its interaction with various cellular processes. However, the intrinsic interplay between autophagy and GC tumorigenesis has not been fully elaborated.

### Tumor suppressor

Several miRNAs are downregulated in GC and act as tumor suppressor through regulating autophagy. Zhao et al. found that miR-29b-3p had a low expression in GC cells compared with normal human gastric mucosal epithelial cells, and that upregulation of miR-29b-3p induced apoptosis by inhibiting the autophagy-related protein MAZ, which can inhibit autophagy by activating the mTORC1 pathway (72). In addition, overexpression of miR-let-7a suppresses cell viability by enhancing autophagy in MGC803 and SGC7901 cells while downregulation of miR-let-7a reverses these effects. Further mechanistic investigations revealed that miR-let-7a regulated cellular autophagic levels by targeting Rictor and further affecting the AKT/mTOR activity (73). A similar study showed that miR-5100 promoted GC cell apoptosis while inhibiting autophagy by targeting caspase activity and apoptosis inhibitor 1, suggesting that downregulated miR-5100 was associated with the poor prognosis of GC patients (74). Coincidentally, high expression of miR-1265 is negatively related to tumor size in GC; moreover, it facilitates apoptosis but suppresses proliferation and autophagy by impairing the LKB1-STRAD-CAB39 complex and regulating the AMPK/mTOR signaling pathway (75). The AKT/mTOR pathway is also activated after upregulating miR-375 in GC cells, and autophagy is subsequently restrained, which inhibits GC cell migration and invasion both *in vitro* and *in vivo* (76). Furthermore, miR-133a-3p is found to target GABARAPL1 to block autophagy-mediated glutaminolysis, thereby repressing GC cell growth and metastasis (77). Similar effects are observed in Epstein-Barr virus-associated GC where miR-BART2-3p is elevated to attenuate autophagy and further reduce epithelial-mesenchymal transition (EMT) and cell migration by targeting ULK1 (78). These findings indicate that tumor-suppressive miRNAs inhibit GC progression by suppressing cytoprotective autophagy *via* targeting autophagy-related components. Additionally, overexpression of miR-133b in GC cells inhibits cell proliferation through eliciting autophagy by regulation of PKM isoforms; however, this inhibitory effect could be partially canceled when cells are administrated with 3-methyladenine, an autophagy inhibitor (79). As a tumor suppressor, miR-140-3p restrains EMT and metastasis in GC cells by suppressing Bcl-2 to further activate beclin-1-dependent autophagy (80). As a GTPase, RAB3IP is involved in the mTOR signaling pathway and regulates autophagy in cancer cells (81). A recent study has shown that miR-532-3p binds to the 3’UTR region of RAB3IP and inhibits its expression, which alleviates RAB3IP-mediated cell proliferation and autophagy inhibition (82). Of interest, H. pylori-induced miR-99b overexpression in GC tissues and BGC823 cells is shown to relieve bacterial loads and block cell proliferation but enhance autophagy by mTOR inhibition (83). Thus, miRNAs also function as tumor suppressor to inhibit GC malignant transformation by activating autophagy.

The downregulated lncRNAs in GC has been clarified to function as tumor suppressors. Mechanistically, these molecules are able to regulate autophagy and further affect GC progression. For example, overexpression of lncRNA FENDRR in GC represses tumor proliferation and invasion through the miR-421/SIRT3/Notch-1 axis (84). Further study revealed that FENDRR deteriorates cell apoptosis but inhibits autophagy *via* epigenetic suppression of ATG7 (85). These results suggest that lncRNAs promote GC cell apoptosis by targeting miRNAs, which may inhibit autophagy. The Notch-1 signaling pathway could be restrained by lncRNA SNHG1 in GC, and the cell proliferation is subsequently suppressed (86). However, depletion of SNHG1 alleviates apoptosis and autophagy by regulating miR-362-3p/JAK2/STAT3 pathway (87). Another deep impressed study demonstrated that lncRNA ADAMTS9-AS2 impeded GC progression and decreased the tumorigenicity of cancer stem cells through regulating SPOP, which may provide a novel target in the treatment of GC (88). Further mechanism analysis showed that ADAMTS9-AS2 downregulated the expression level of p62 and antiapoptotic protein Bcl-2 while upregulating the expression level of LC3-II, beclin-1, and the proapoptotic protein Bax by inactivating the PI3K/AKT/mTOR signaling pathway (89). In addition, upregulation of lncRNA DRAIC attenuates GC proliferation and metastasis *via* impairing the combination of UCHL5 and NFRKB and mediating the ubiquitination degradation of NFRKB (90). As a tumor suppressor, DRAIC can induce autophagy to maintain the cancer malignant phenotype *via* activating the AMPK-mTOR-S6K/ULK1 pathway (91). Therefore, lncRNAs seem to suppress GC progression by promoting autophagy, but there is lacking of direct evidence to support this point.

Also, low expressed circRNAs in GC have been implicated in tumor suppressor through regulating autophagy. Zhu et al. investigated the inhibitory effects of circRNA_KIAA0907 on migration and proliferation of GC cells. In their study, a remarkable KIAA0907 low expression was observed in GC compared with their adjacent normal tissue. KIAA0907 overexpression inhibited the cell cycle, proliferation, and autophagy, as well as promoted apoptosis. Molecular investigation demonstrated that KIAA0907 functioned as a specific sponge for miR-452-5p and further accelerated the expression of lysine acetyltransferase 6B. For *in vivo* efficacy assessment, a mouse GC model was used. KIAA0907 overexpression repressed proliferation of GC in mice. They also indicated that upregulation of KIAA0907 suppressed cell proliferation (92). Thus, this study demonstrates that circRNAs promote GC cell apoptosis but inhibit autophagy by targeting miRNAs. However, autophagy activation is also associated with the tumor-suppressive effect of circRNAs. A recent study has showed that circRNA-ST3GAL6 inhibits the malignant behaviors of GC by inducing apoptosis and autophagy through miR-300/FOXP2 axis (93). Likewise, circRNA-RELL1 blocks GC cell proliferation, invasion, migration *via* autophagy activation through the miR-637/EPHB3 axis (94).

In conclusion, various tumor suppressive ncRNAs are downregulated in GC cells compared with normal ones. Overexpression of these ncRNAs can inhibit cell proliferation and malignant transformation, accompanied with increased or reduced levels of autophagy. Modulation of autophagy by these ncRNAs is complicated due to they both target autophagy-related signaling pathways and ATGs (**Table 2**). On most occasions, tumor-suppressive ncRNAs inhibit cytoprotective autophagy. However, ncRNAs also induce pro-death autophagy to inhibit GC progression (80, 91). Overexpression of some tumor suppressor ncRNAs to block autophagy has become a potential therapeutic strategy in cancer *via* different molecular pathways (95). Thus, upregulation of tumor suppressive ncRNAs may be beneficial for GC treatment by inhibiting protective autophagy or facilitating autophagic cell death. Otherwise, autophagy induces cell death by crosstalk with non-apoptotic cell death pathways (96). It can be hypothesized that when tumor-suppressive ncRNAs activate nonapoptotic signals to provoke GC cell death, autophagy regulation may serve as an adaptive response to participate in ncRNAs-mediated tumor suppression. Thus, further clarifying the role of ncRNA-regulating autophagy is vital for GC treatment. In addition to ncRNAs dysfunction, the fluctuant autophagy level during GC progression may be associated with various stages of tumor development, histological and molecular subtypes of tumor, and other conditions.

**Table 2:** NcRNAs serve as tumor suppressors in GC.

NcRNA	Expression	Targets	Autophagy	Outcome	Experimental model	Ref.
miR-29b-3p	Downregulated	MAZ↓→mTOR↓→beclin-1↑, LC3-II↑, p62↓	Activated	Inhibit proliferation, migration and invasion, promote apoptosis	AGS, MGC803 cells; nude mice	(72)
miR-let-7a	Downregulated	mTOR complex 2↓→LC3, p62↑	Activated	Inhibit cell proliferation, migration, and invasion	MGC803 and SGC7901 cells	(73)
miR-5100	Downregulated	CAAP1↓→LC3-II ↓	Inhibited	Enhance apoptosis	MGC80-3, AGS, SGC-7901 cells; nude mice; human GC tissues	(74)
miR-1265	Downregulated	CAB39↓- AMPK↓, mTOR↑→beclin-1↑, LC3B↓, p62↑	Inhibited	Suppresses proliferation and induces apoptosis	MKN45, SGC7901 cells; human GC tissues	(75)
miR-375	Downregulated	AKT/ mTOR↑→LC3-II↓, ATG 7↓	Inhibited	Suppress proliferation, EMT, invasion and migration	MKN45, GT3TKB cells; nude mice	(76)
miR-133a-3p	Downregulated	ULK↓, Beclin-1↓, LC3-II↓, ATG 5↓, p62↑; ATG 7↓, ATG13↓, ATG8↓,	Inhibited	Inhibit proliferation, EMT, migration and invasion	BGC823, SGC7901, MGC803, MKN45, HGC27, AGS cells; nude mice; human GC tissues	(77)
miR-133b	Downregulated	PTBP1↓→PKM2/PKM1↓→LC3-II↑, beclin-1↑	Activated	Inhibit cell and tumor growth	MKN1, MKN45 and KATOIII cells; human GC tissues	(79)
miR-140-3p	Downregulated	Bcl-2↓→LC3B↑, beclin-1↑, p62↓	Activated	Restrain migration, invasion, EMT and metastasis	BGC823, MKN45, MKN28, MGC803, and SGC7901 cells; nude mice; human GC tissues	(80)
miR-532-3p	Downregulated	Rab3IP↓→mTOR↓→LC3-II↑, p62↓	Activated	Inhibit proliferation	AGS, MKN45, BGC823, SGC7901, MGC803, and MKN28 cells; human GC tissues	(82)
CircRNA_ KIAA0907	Downregulated	miR-452-5p↓→KAT6B↑→Bax/Bcl-2↓→ beclin-1↓, LC3-II↓, p62↑	Inhibited	Inhibit proliferation, cell cycle, reduce apoptosis	HGC27, AGS, MKN45, and NCIN87 cells; nude mice; human GC tissues	(92)
CircRNA_ST3GAL6	Downregulated	miR-300↓→FOXP2↑→mTOR↓→LC3-II↑, p62↓	Activated	Inhibit growth and metastasis	MGC803, BGC823, MKN45, SGC7901 cells; nude mice; human GC tissues	(93)
CircRNA_ RELL1	Downregulated	miR-637↓→EPHB3↑→p62↓	Activated	Inhibit proliferation, growth and metastasis	AGS, SGC7901, MKN45, and MGC823 cells; nude mice; human GC tissues	(94)

↑, upregulation; ↓, downregulation.

### Chemoresistance

Series of miRNAs are upregulated in GC and promote chemoresistance by regulating autophagy. A recent study has revealed that miR-3174 is highly expressed in GC cells and inhibits autophagic cell death by the ARHGAP10/mTORC1 axis, thus leading to cisplatin resistance (97). In another study performed by Gu et al., autophagy induction sensitizes GC cells to cisplatin, whereas its inhibition yields the opposite effects. Compared to parent cells, cisplatin-resistant GC cells express higher levels of miR-21. In GC cells transfected with miR-21 mimics, cisplatin resistance is restored *via* inhibiting autophagy by the PI3K/AKT/mTOR pathway; however, miR-21 suppressors confer GC cells to cisplatin sensitivity through activation of autophagy (98). These findings demonstrate that autophagy induction can be an effective therapeutic strategy in cisplatin-resistant GC. However, autophagy can counteract the chemotherapy-induced oxidative stress reaction. Oxidative stress-induced damage to cancer cells is a main model of action by chemotherapeutic drugs. Wang et al. showed that oxaliplatin-mediated oxidative stress could activate lncRNA NORAD, which further sponges miR-433-3p and stabilizes the ATG5-ATG12 complex, thereby enhancing the autophagy flux to alleviate oxidative stress, and ultimately leading to oxaliplatin resistance in GC (99). Thus, suppression of autophagy may be beneficial to inhibit oxaliplatin resistance in this context.

Some lncRNAs are overexpressed in GC tissues and induce cell resistance to the growth inhibition and apoptosis, which are mediated by chemotherapeutic agents through regulating autophagy. For instance, upregulated lncRNA FEZF1-AS1 in chemoresistant GC tissues drives chemoresistance of cancer cells to 5-FU through facilitating autophagy by directly targeting ATG5 (100). Similarly, lncRNA HULC, which indicates a poor prognosis in GC patients, induces autophagy and DDP resistance by inhibiting the ubiquitination of FoxM1 (101). Thus, silencing of lncRNA HULC elicits GC cell apoptosis induced by cisplatin (102). In addition, LINC00963, a new drug-resistant lncRNA, enhances proliferation and migration in oxaliplatin-resistant cells by negatively regulating miR-4458. Both downregulation of LINC00963 and upregulation of miR-4458 suppress autophagy by reducing the expression of ATG16, which indicates that targeting LINC00963 to block autophagy sensitizes GC cells to oxaliplatin (103). The miR188-3p/ATG14 axis is also targeted by lncRNA EIF3J-DT to activate autophagy and induce multiple chemotherapeutic drug resistance in GC cells (104). In cisplatin-resistant GC cells, LINC01572 is upregulated to induce chemoresistance, while depletion of LINC01572 promotes cisplatin sensitivity by inhibiting drug-induced autophagy through the miR-497-5p/ATG14 regulatory axis (105). A similar study unveiled that highly expressed LINC00641 in GC cells mediates oxaliplatin resistance *via* targeting miR-582-5p to regulate autophagy process (106). Besides, lncRNA MALAT1 functions as a ceRNA for miR-30b to sequester miR-30b from ATG5, thus inducing autophagy and cisplatin resistance (107). Of interest, MALAT1 causes poor disease-free survival and overall survival in patients who have received 5-FU-based adjuvant therapy; moreover, this lncRNA induces the resistance of GC cells to cisplatin, 5-FU and vincristine by triggering autophagy through sequestration of miR-23b-3p and then elevating ATG12 levels (108). Further mechanistic evaluation revealed that MALAT1 promotes GC cell proliferation and cisplatin resistance *via* the PI3K/AKT pathway (109). However, whether MALAT1-induced cisplatin resistance and autophagy is regulated by the PI3K/AKT pathway remains elusive. Additionally, overexpressed lncRNA HOTTIP in cisplatin-resistant GC cell line restrains autophagy and induces chemoresistance through sponging miR-216a-5p and further upregulating the Bcl-2 expression but decreasing the beclin-1 expression (110). These findings indicate that overexpressed lncRNAs in GC induce cell chemoresistance by inducing autophagy. However, when apoptosis is inhibited by lncRNAs to make GC cells become resistant to chemotherapeutic agents, autophagy might be suppressed as an additional effect.

CircRNAs are also upregulated in GC cells and contribute to chemoresistance by regulating autophagy. Yao and co-workers conducted a study on chemotherapeutic implication of circRNA_PVT1 in cisplatin-resistant GC cells. They showed that the knockdown of PVT1 expedited cisplatin sensitivity of GC *via* inhibiting autophagy and further promoting apoptosis and decreasing invasion. Molecular investigation demonstrated that PVT1 promoted cisplatin resistance and autophagy *via* the miR-30a-5p/YAP1 axis (111). Likewise, Ma et al. investigated the effects of miR-375 on circRNA_CGAP1-mediated apatinib chemoresistance of GC cells. In their study, a remarkable miR-3657 low expression was observed in GC tissues in comparison with their corresponding paracarcinoma samples. Silencing of CGAP1 inhibited apatinib-induced autophagy by stabilizing miR-3657 to augment its suppression on ATG7 expression; moreover, knockdown of CGAP1 sensitized GC cells to apatinib *via* autophagy inhibition *in vitro* and *in vivo* (112), suggesting that targeting CGAP1 may provide a novel target in the treatment of cisplatin-resistant GC. Also, overexpressed circRNA-CPM leads to the activation of autophagy and 5-FU chemoresistance in GC cells and tissues *via* the miR-21-3p/PRKAA2 axis (113). These results imply that overexpressed circRNAs in GC mediate chemoresistance by inducing autophagy, and that suppression of autophagy could improve chemotherapy efficacy in GC.

Collectively, chemoresistance-related ncRNAs are highly expressed in GC tissues and mediate the resistance to various chemotherapeutic drugs, along with various levels of autophagy. Of importance, most ncRNAs are associated with increased levels of autophagy, especially when ncRNAs directly upregulate ATGs (**Table 3**). Silencing these ncRNAs may alleviate the resistance of GC cells to chemotherapeutic agents by reducing protective autophagy. In fact, genetic interventions targeting ncRNAs such as miRNA mimics, miRNA sponges, anti-miRNA oligonucleotides are useful approaches to combat chemoresistance by regulating autophagy in cancer treatment (114). Thus, chemotherapies combined with suppression of autophagy *via* downregulating these ncRNAs or targeting ATGs may provide a promising therapeutic strategy for GC.

**Table 3:** Chemoresistance-related ncRNAs in GC.

NcRNA	Expression	Targets	Autophagy	Outcome	Experimental model	Ref.
miR-21	Upregulated	PI3K/AKT/mTOR↑→ beclin-1↓, LC3-II↓	Inhibited	Cisplatin resistance	AGS cells	(98)
LncRNA NORAD	Upregulated	miR-433-3p↓→ ATG5↑, ATG12↑	Activated	Oxaliplatin resistance	SGC7901 cells	(99)
LncRNA FEZF1-AS1	Upregulated	LC3-II↑, ATG5↑	Activated	5-fluorouracil resistance	MKN49P, MGC803, BGC823, SGC7901, and NCIN87 cells; nude mice; human GC tissues	(100)
LncRNA HULC	Upregulated	FoxM1↓→LC3-II↑, beclin-1↑, p62↓	Activated	Cisplatin resistance	SGC7901 and MGC-803 cells; nude mice	(101)
LINC00963	Upregulated	miR-4458↓→ATG16L1 ↑	Activated	Oxaliplatin resistance	SGC-7901, MKN45 and MKN74 cells; nude mice; human GC tissues	(103)
LncRNA EIF3J-DT	Upregulated	miR188-3p↓→ATG14↑	Activated	Oxaliplatin and 5-fluorouracil resistance	MGC803, MKN45 cells; nude mice; human GC tissues	(104)
LINC01572	Upregulated	miR-497-5p↓→ ATG14↑	Activated	Cisplatin resistance	BGC823 and SGC7901 cells; nude mice; human GC tissues	(105)
LINC00641	Upregulated	miR-582-5p↓→LC3-II↑, p62↓	Activated	Cisplatin resistance	MKN45, SGC7901 and MKN28 cells; human GC tissues	(106)
LncRNA MALAT1	Upregulated	miR-30b↓→ATG5↑	Activated	Cisplatin resistance	AGS, HGC27 and 293T cells	(107)
LncRNA MALAT1	Upregulated	miR-23b-3p↓→ ATG12↑	Activated	5-fluorouracil, vincristine and cisplatin resistance	SGC7901 and BGC823 cells; nude mice; human GC tissues	(108)
LncRNA HOTTIP	Upregulated	miR-216a-5p↓→Bcl-2↑→beclin1↓	Inhibited	Cisplatin resistance	SGC7901 cells; nude mice; human GC tissues	(110)
CircRNA_PVT1	Upregulated	miR-30a-5p↓→YAP1↑→LC3-II↑, p62↓	Activated	Cisplatin resistance	HGC27 and AGS cells; nude mice; human GC serum	(111)
CircRNA_CGAP1	Upregulated	miR-3657↓→ATG7↑	Activated	Apatinib resistance	BGC823 and HGC27 cells; nude mice; human GC tissues	(112)
CircRNA_CPM	Upregulated	miR-21-3p↓→PRKAA2↑→LC3-II↑, p62↓	Activated	5-fluorouracil resistance	AGS and HGC‐27 cells; nude mice; human GC tissues	(113)

↑, upregulation; ↓, downregulation.

### Chemosensitivity

Of importance, increasing researches have emphasized the role of miRNAs on enhancing the sensitivity of chemotherapeutic drugs in GC, in which the autophagic process is involved. MiR-181b-5p, regulated by the transcription factor CAGE, is downregulated in chemoresistant GC cells and reduces autophagic flux to promote the sensitivity to various anticancer drugs in these cells by targeting S1PR1 and further inhibiting the expression of beclin-1 (115). Similarly, overexpression of miR-495-3p is sufficient to reverse the multidrug resistant-cell to four chemotherapeutics and suppress the GC tumor growth, as miR-495-3p attenuates the process of autophagy *via* binding to GRP78 and thus activating mTOR (116). The PI3K/AKT/mTOR pathway is also regulated by miR-361-5p in GC to block autophagy and further to inhibit chemoresistance to docetaxel *in vitro* (117). In addition, upregulation of miR-181a in cisplatin-resistant SGC7901 cells expedites cisplatin sensitivity and reduces the growth of GC xenografts *in vivo* through negative regulating autophagy *via* targeting ATG5 (118). Similar to scenario observed in these cells, Du et al. have reported that miR-30a elevates the expression of P-gp and MDR1, reduces the expression of LC3-II, impedes autophagy, and reverses cisplatin resistance (119). Also, the overexpressed miR-874 in GC cells counteracts the multidrug resistance by directly downregulating the expression of ATG16L1 and thus blocking autophagy (18). Intriguingly, miR-148a-3p decreases the expression of A-kinase anchoring protein 1 and RAB12, a member of RAS oncogene family, and alleviates the inhibitory effects of RAB12 on mTORC1, thereby suppressing autophagy and reversing the resistance of GC cells to cisplatin (120). Of importance, miR-143 is regarded as an autophagy inhibitor to improve the efficacy of quercetin in GC cells *via* targeting GABARAPL1 (121). Moreover, miR-23b-3p is shown to inhibit autophagy, and reverses GC cell resistance to cisplatin, vincristine, and 5-FU by directly targeting ATG12 (122).These findings suggest that miRNAs induce chemosensitivity in GC though inhibiting autophagy. Thus, in GC with these downregulated miRNAs, chemotherapeutic drugs combined with autophagy inhibitors might provide a potential therapeutic strategy for this disease.

Additionally, lncRNAs have been demonstrated to be associated with chemosensitivity in GC by regulating autophagy. In a study performed by Zhang et al., decreased lncRNA CRNDE expression was related to the chemoresistance in GC cells, whereas its upregulation exhibited opposite effects. Further mechanistic investigations revealed that CRNDE increased proteasome ubiquitination-dependent degradation of SRSF6, a classical splicing factor. They unveiled that SRSF6 enhanced GC cellular autophagy by regulating alterative splicing of PICALM (S-to-L isoform switch), thereby mediating autophagy-induced resistance to oxaliplatin and 5-FU. Their results suggested that autophagy inhibited by the CRNDE/SRSF6 axis could be an effective therapeutic approach in chemoresistant GC (123). This finding implies that lncRNA CRNDE enhances chemosensitivity through suppressing autophagy. However, CRNDE derived from tumor-associated macrophages is demonstrated to promote GC cells proliferation and cisplatin resistance (124). The overexpressed CRNDE indicates a poor prognosis in GC patients; moreover, depression of CRNDE attenuates GC cell proliferation, migration and invasion by targeting miR-145 and affecting PI3K/AKT signaling pathways (125, 126). Therefore, the role of lncRNA CRNDE in GC is dependent on different cell types and conditions.

Furthermore, circRNAs can promote chemosensitivity in GC *via* autophagy regulation. Sun et al. investigated the effects of circRNA_MCTP2 on efficacy of chemotherapeutic agents in GC cells. In their study, a remarkable MCTP2 low expression was observed in cisplatin-resistant GC cells and tissues. MCTP2 overexpression inhibited proliferation and promoted apoptosis in GC cells. Most importantly, MCTP2 suppressed autophagy and sensitized GC cells to cisplatin by sponging miR-99a-5p and further upregulating the expression of MTMR3. For *in vivo* efficacy assessment, a GC mouse model was used. MCTP2 overexpression alleviated cisplatin resistance *in vivo*. They also proposed that upregulation of MCTP2 could be a promising therapeutic strategy for counteracting cisplatin resistance in GC (127). Similarly, overexpression of circRNA_CUL2 in cisplatin-resistant AGS and SGC7901 GC cells repressed malignant transformation *in vitro* and tumorigenicity *in vivo*, as CUL2 modulated tumor progression by sponging miR-142-3p to regulate ROCK2. Further investigation revealed that CUL2 enhanced the sensitivity of GC cells to cisplatin through miR-142-3p/ROCK2-mediated autophagy activation (128), since ROCK2 inhibition was associated with autophagy induction (129, 130). These results suggest that circRNAs can promote chemosensitivity in GC through modulating autophagy.

In sum, the expression level of chemosensitivity-related ncRNAs is depressed in GC tissues, and upregulation of these ncRNAs sensitizes GC cells to various chemotherapeutic drugs through inhibiting autophagy by both regulating autophagy-related signaling pathways and targeting ATGs (**Table 4**). The levels of some ncRNAs are negatively correlated with protective autophagy and drug resistance following chemotherapy, therefore rejuvenation of these downregulated ncRNAs in drug resistant GC cells may restore chemosensitivity by inhibiting autophagy. Moreover, ncRNA-based therapeutic approaches, including ncRNA-coated nanoparticles and ncRNA microinjection, are developed to enhance chemosensitivity (131). In this context, inhibition of cytoprotective autophagy by ncRNAs may pave the way to combat chemoresistance.

**Table 4:** Chemosensitivity-related ncRNAs in GC.

NcRNA	Expression	Targets	Autophagy	Outcome	Experimental model	Ref.
miR-181b-5p	Downregulated	S1PR1↓→ ATG5↓, beclin1↓, LC3-II↓, p62↑	Inhibited	Celastrol, taxol, doxorubicin and docetaxel sensitivity	AGS cells	(115)
miR-495-3p	Downregulated	GRP78↓→mTOR↑→LC3-II↓, p62↑	Inhibited	Vincristine and adriamycin sensitivity	SGC7901 cells, nude mice, human GC tissues	(116)
miR-361-5p	Downregulated	PI3K/AKT/mTOR↑→LC3-II↓, beclin-1↓, p62↑	Inhibited	Docetaxel sensitivity	SGC7901 and MKN28 cells	(117)
miR-181a	Downregulated	ATG5↓	Inhibited	Cisplatin sensitivity	SGC7901 cells and nude mice	(118)
miR-30	Downregulated	LC3-II↓	Inhibited	Cisplatin sensitivity	SGC7901 cells	(119)
miR-874	Downregulated	ATG16L1↓, LC3-II↓	Inhibited	Cisplatin sensitivity	SGC7901 and BGC823 cells, nude mice, human GC tissues	(120)
miR-148a-3p	Downregulated	RAB12↓→ mTOR1↑→LC3-II↓, beclin-1↓, p62↑	Inhibited	Cisplatin sensitivity	SGC7901 and BGC823 cells, nude mice, human GC tissues	(121)
miR-143	Downregulated	LC3-II↓, beclin-1↓	Inhibited	Quercetin sensitivity	AGS and MKN28 cells, human GC tissues	(122)
miR-23b-3p	Downregulated	ATG12↓, HMGB2↓→LC3-II↓, p62↑	Inhibited	5-fluorouracil, vincristine and cisplatin sensitivity	SGC7901, AGS and BGC823 cells; nude mice; human GC tissues	(123)
LncRNA CRNDE	Downregulated	SRSF6↓→LC3-II↓, p62↑	Inhibited	5-fluorouracil and cisplatin sensitivity	MGC803 cells; nude mice; human GC tissues	(124)
CircRNA_ MCTP2	Downregulated	miR-99a-5p↓→MTMR3↑→LC3-II↓, beclin-1↓, p62↑	Inhibited	Cisplatin sensitivity	BGC823 and SGC7901 cells; nude mice; human GC tissues	(127)
CircRNA_CUL2	Downregulated	miR-142-3p↓→ROCK2↑→LC3-II↓, beclin-1↓, p62↑	Inhibited	Cisplatin sensitivity	AGS, SGC7901, MKN45, and BGC823 cells; nude mice; human GC tissues	(128)

↑, upregulation; ↓, downregulation.

## Conclusions

In this review, the role of ncRNAs-induced autophagy in the malignant transformation and drug resistance of GC are summarized in detail. NcRNAs are shown to serve as both oncogenes and tumor suppressors in GC. Of interest, NcRNA-regulated autophagy also perform a dual function in the progression and chemotherapy of GC. How the autophagy mechanism exerts a dual effect in the GC is unclear. It can be postulated that the role of autophagy is determined by its upstream modulators. Whether oncogenic ncRNAs or tumor-suppressive ncRNAs is dependent on various levels and functions of autophagy to affect GC progression and chemotherapeutic efficacy. Briefly, as tumor suppressors, ncRNAs inhibit cytoprotective autophagy during cainogenesis or induce autophagic cell death at the advanced stages of GC; whereas oncogenic ncRNAs trigger cytoprotective autophagy to facilitate tumorigenesis and progression of GC. The regulatory mechanisms of ncRNAs on autophagy is intricate. Noteworthy, oncogenic ncRNAs tends to regulate autophagy-related signaling pathways to inhibit autophagic cell death, and chemoresistance-related ncRNAs mainly increase the expression of ATGs to activate cytoprotective autophagy; moreover, upregulating the expression of ncRNAs that is originally repressed in tumor tissues promote tumor suppression and chemosensitivity through autophagy modulation *via* both regulating autophagy-related signaling pathways and ATGs. In this context, targeting oncogenic ncRNAs combined with autophagy inhibitors can be employed as a promising strategy to promote GC cells death and to sensitize them to chemotherapy. Thus, clarification of the regulatory role of ncRNAs on autophagy in GC development and chemoresistance is expected to strategize beneficial options in ncRNA-based therapies to eradicate this disease, as it will help contribute towards addressing key issues in GC drug resistance.

## Author contributions

ZW wrote the manuscript. JL, JX, XY, and BW contributed to the writing and drafted the figures. WS and YZ critically reviewed the manuscript and contributed to the writing. All authors contributed to the article and approved the submitted version.

## Funding

This work is supported by the National Natural Science Foundation of China (81973601), TCM research projects of Heilongjiang Province (ZHY2020-041) and Postdoctoral funding of Heilongjiang Province (LBH-Z21218).

## Conflict of interest

The authors declare that the research was conducted in the absence of any commercial or financial relationships that could be construed as a potential conflict of interest.

## Publisher’s note

All claims expressed in this article are solely those of the authors and do not necessarily represent those of their affiliated organizations, or those of the publisher, the editors and the reviewers. Any product that may be evaluated in this article, or claim that may be made by its manufacturer, is not guaranteed or endorsed by the publisher.

## Abbreviations

AMPK, adenosine monophosphate-activated protein kinase; miRNA, microRNA; AMBRA, autophagy and beclin-1 regulator; MAPK, mitogen activated kinase-like protein; ATGs, autophagy-related genes; ncRNA, non-coding RNA; CAAP1, caspase activity and apoptosis inhibitor 1; PI3K, phosphatidylinositol 3-kinase; ceRNA, competing endogenous RNA; PI3P. phosphatidylinositol 3-phosphate; circRNA, circular RNA; PAS, phosphatidylethanolamine; ERK, extracellular signal-regulated kinase; PE, pre-autophagosomal structure EMT; epithelial-mesenchymal transition PTEN phosphatase and tensin homolog ER endoplasmic reticulum TSC Tuberous sclerosis complex GC gastric cancer ULK1 unc-51 like autophagy activating kinase JNK c-Jun N-terminal kinase UVRAG, ultraviolet radiation resistance-associated gene; LC3, microtubule associated protein light chain 3; VPS15, vacuolar protein sorting 15; LncRNA, long non-coding RNA; WIPI2, WD repeat domain phosphoinositide-interacting protein; mTORC, mammalian target of rapamycin; ZFYVE1, zinc-finger FYVE domain-containing protein 1.
